# Real-World Infective Endocarditis in a Regional Hospital: Clinical Severity, Guideline Adherence, and Determinants of In-Hospital Outcomes

**DOI:** 10.3390/jcm15103600

**Published:** 2026-05-08

**Authors:** Călin Pop, Lucian Liviu Pop, Maria Rebeca Petruș, Andreea Ioana Talpos, Roxana Hodas, Lavinia Pop, Iulia Pop

**Affiliations:** 1 Department of Cardiology, “Constantin Opriş” Emergency County Hospital, Str. George Cosbuc Nr. 31, 430130 Baia Mare, Romania; secr.cardiologie@sjbm.ro (M.R.P.); cardiologie@sjbm.ro (A.I.T.); roxana.hodas@yahoo.com (R.H.); laviniapop10@yahoo.com (L.P.); dr_iulia_muresan@yahoo.com (I.P.); 2 Faculty of Nursing and Health Sciences, University of Medicine and Pharmacy “Iuliu Hațieganu”, Str. Louis Pasteur Nr. 4, 400349 Cluj-Napoca, Romania; pop.lucianliviu@yahoo.co

**Keywords:** infective endocarditis, real-world evidence, in-hospital mortality, prognostic factors, guideline adherence, trial eligibility, observational study, microbiology, complications

## Abstract

**Background and Objectives:** Infective endocarditis (IE) remains associated with high mortality, and real-world (RW) patients often differ from trial populations. We evaluated predictors of complications and mortality, the trial-eligibility gap, and temporal trends in guideline adherence across two periods (Period 1 [P1]: 2011–2016 vs. Period 2 [P2]: 2017–2025) in a Romanian county hospital. **Materials and Methods:** We conducted a retrospective analysis of consecutive adult patients with definite IE. Patients were categorized as trial-eligible (TE) or RW according to predefined criteria. The composite endpoint included acute heart failure, cardiogenic or septic shock, embolic events, infectious complications, need for renal replacement therapy, and in-hospital mortality. We evaluated guideline adherence using a predefined quality indicator (QI) score ≥ 3. We identified independent predictors of outcome using multivariable logistic regression. **Results:** Among 206 patients (mean age 63.0 ± 14.8 years; 70.4% male), blood cultures were positive in 64.1%, with *Staphylococcus aureus* accounting for 14.1%. Vegetations were documented in 72.8%, and cardiac surgery was performed in 26.2%. Overall, at least one event from the composite endpoint occurred in 61.6%, and mortality was 32.5%. TE patients represented 63.1% of the cohort. Guideline adherence improved over time (QI ≥ 3: from 18.3% in P1 to 25.4% in P2, *p* = 0.32). In P2, the composite endpoint (66.8% vs. 42.9%, *p* = 0.002) and embolic events (31.8% vs. 8.2%, *p* < 0.001) were more frequent, whereas mortality remained unchanged (31.8% vs. 34.7%, *p* = 0.844). Sepsis at admission and left ventricular ejection fraction (LVEF) < 50% independently predicted adverse outcomes; model discrimination was acceptable, with an area under the curve (AUC) of 0.77. **Conclusions:** RW IE showed high complication rates and a persistent trial gap; improved guideline adherence was offset by greater clinical severity.

## 1. Introduction

IE remains a severe condition with high morbidity and mortality despite advances in diagnostics, antimicrobial therapy, and cardiac surgery. Contemporary data report an incidence of 10–15 cases per 100,000 person-years, with in-hospital mortality exceeding 15–30% and one-year mortality approaching 40% [1,2].

The introduction of structured management strategies has improved the standardization of care. The 2015 European Society of Cardiology (ESC) guidelines defined key QIs, including appropriate blood cultures, multimodal imaging, multidisciplinary evaluation, and timely surgery [3]. The 2023 update placed further emphasis on endocarditis teams and advanced imaging, although many recommendations still rely on limited evidence [1,3,4]. In this context, observational studies are essential to evaluate RW implementation. Large registries have documented important epidemiological shifts, including aging populations, increased comorbidity burden, and a growing proportion of *Staphylococcus aureus*, enterococcal, and prosthetic or device-related infections [2,5,6,7]. Registry-based efforts, such as a nationwide consortium of endocarditis teams (ENDOCOR) in the Netherlands, illustrate attempts to integrate systematic data collection and quality monitoring into clinical workflows [8]. However, most data come from tertiary centers, whereas regional hospitals, particularly in Eastern Europe, remain underrepresented despite known differences in case mix, access to surgery, and diagnostic resources [2,6].

An additional challenge is the gap between randomized controlled trial (RCT) populations and RW patients. Studies on partial oral therapy illustrate this discrepancy, as TE populations are highly selected, whereas many routine patients would not meet eligibility criteria despite acceptable outcomes [9,10,11]. This “trial eligibility gap” reflects the exclusion of elderly, frail, or comorbid patients, as well as those with prosthetic valves or right-sided IE, who are commonly encountered in routine practice [6,9,12,13].

Romanian data provide valuable but partial insights, as relatively low surgical intervention rates and in-hospital mortality exceeding one-third of cases mainly originate from tertiary centers or specific subgroups such as prosthetic valve IE or injection drug use (PWID) [14,15,16,17,18]. Consequently, the broader spectrum of IE encountered in regional hospitals remains insufficiently characterized.

Several gaps persist, including limited data on the proportion of TE vs. RW patients, adherence to guideline-based QIs, and temporal trends in routine care. These aspects are particularly relevant for county hospitals, which manage unselected populations and must coordinate care in settings with variable access to cardiac surgery and multidisciplinary infrastructure. The period from 2011 to 2025 includes major developments in IE management, such as expanded use of advanced imaging, structured multidisciplinary care, and evolving therapeutic strategies [1,3,4,6]. Evaluating their RW implementation and impact at the regional level is therefore clinically relevant.

The objectives of this study were: (i) to describe the clinical, microbiological, and echocardiographic features of adults with definite IE treated at a Romanian county hospital from 2011 to 2025; (ii) to distinguish between patients who meet contemporary RCT eligibility criteria and those who represent RW practice; (iii) to identify factors associated with a composite endpoint that includes acute heart failure, shock, major embolic events, severe infectious complications, renal replacement therapy, and/or in-hospital death; and (iv) to assess temporal changes in guideline-concordant management and QIs by comparing two time intervals (P1: 2011–2016 vs. P2: 2017–2025). By contextualizing county-level data within guideline standards and RCT eligibility, this study aims to complement existing evidence and highlight practical considerations for improving IE care in similar settings. 

## 2. Methods

### 2.1. Study Design

This retrospective, single-center observational cohort study included adult patients diagnosed with definite IE. The research took place in the Department of Cardiology and Cardiovascular Intensive Care at the “Dr. Constantin Opriș” Emergency County Hospital in Baia Mare, Romania, a secondary/tertiary referral institution that provides interventional cardiology services at the regional level. The study was divided into two predefined periods, P1: 1 January 2011–31 December 2016 and P2: 1 January 2017–31 December 2025, to reflect major changes in diagnostic and management practices. After 2016, transesophageal echocardiography (TEE) was implemented systematically, microbiological workup became more standardized, and an on-site cardiac surgery program became operational in 2023. These changes improved diagnostic accuracy and consistency of care. The Institutional Review Ethics Committee of “Dr. Constantin Opriș” Emergency County Hospital, Baia Mare, reviewed and approved the study protocol on 2 February 2026 (decision 4979). Given the retrospective design, the use of routinely collected clinical data, and prior anonymization, the requirement for individual informed consent was waived in accordance with national regulations and institutional policies. During manuscript preparation, the authors used ChatGPT-5.5 to create the graphical abstract. The study adhered to the STrengthening the Reporting of OBservational studies in Epidemiology (STROBE) guidelines for cohort and observational studies [19].

### 2.2. Study Population

All consecutive adults (≥18 years) hospitalized with suspected IE during the study period were screened. Inclusion required a definite IE diagnosis according to the modified Duke criteria (ESC 2015) and the updated ESC 2023 definitions [1,3,20]. For patients with multiple admissions, only the index episode was included. Inclusion criteria were age ≥ 18 years, a definite IE diagnosis, and the availability of clinical, microbiological, and echocardiographic data. Exclusion criteria included possible or rejected IE diagnosis, transfer in or out within 24 h with insufficient data, and isolated catheter-related right-sided infections without essential diagnostic information. Follow-up was limited to the index hospitalization, as the study focused on in-hospital outcomes. No systematic post-discharge follow-up data were available in this retrospective dataset.

### 2.3. Variables and Data Sources

Data were abstracted from digital records, microbiology databases, echocardiography archives, and discharge documentation. Variables were grouped into predefined domains. Demographics and comorbidity burden included age, sex, and a global comorbidity burden defined as the cumulative number of major chronic conditions [hypertension (HTN), diabetes mellitus (DM), chronic heart failure (HF), chronic kidney disease (CKD), malignancy, liver cirrhosis, and coronary artery disease (CAD)]. Clinical presentation and severity variables included symptom duration, fever, acute heart failure, sepsis at admission, requirement for vasopressors, and need for invasive mechanical ventilation. Microbiological variables included the number and timing of blood cultures, causative pathogens (with specific categorization of *Staphylococcus aureus*, *Enterococcus* spp., and *Streptococcus viridans*), and culture-negative IE. Imaging and anatomical variables included the timing of transthoracic echocardiography (TTE) and TEE, valve involvement (native vs. prosthetic), right-sided involvement, vegetation size ≥ 10 mm, severe valvular regurgitation, perivalvular complications (abscess or fistula), and LVEF < 50%. Management variables included antimicrobial regimens, timing of surgery, patient transfers, and multidisciplinary evaluations. Outcome variables included a composite endpoint, in-hospital mortality, renal replacement therapy, embolic events, and length of hospital stay. Two independent investigators performed case selection and data validation, and a senior cardiologist adjudicated discrepancies.

### 2.4. Definitions and Endpoints

Cardiogenic shock (CS) was defined according to the Shock Academic Research Consortium (SHARC) expert panel criteria [21]. Septic shock was defined based on the Sepsis-3 criteria [22]. Sepsis at admission was defined according to the Sepsis-3 criteria as suspected or confirmed infection associated with acute organ dysfunction (increase in SOFA score ≥ 2 points) present at hospital presentation. Complete SOFA variables were not available in a subset of retrospective cases (approximately 15%), mainly in the earlier study period (P1), where some laboratory or clinical parameters were not systematically recorded. In cases where complete SOFA variables were unavailable, clinically documented organ dysfunction (hypotension, acute kidney injury, respiratory insufficiency, altered mental status, thrombocytopenia, or elevated lactate) was considered consistent with sepsis [22]. The primary outcome was a composite in-hospital endpoint, defined as the occurrence of at least one of the following events: acute heart failure that required intravenous therapy or ventilatory support; shock (cardiogenic and/or septic); embolic events that were symptomatic or confirmed by imaging or surgery; severe infectious complications (such as abscess, fistula, or metastatic infection); renal replacement therapy; and all-cause in-hospital mortality. Secondary outcomes included analysis of individual components and length of hospital stay.

### 2.5. Classification of TE vs. RW Patients

To evaluate the gap between RCT-eligible and routine-practice populations, patients were classified as TE if they fulfilled predefined clinical stability and selection criteria derived from contemporary IE trials and guideline-based frameworks [1,3,4,9]. Eligibility required the absence of the following conditions: age > 80 years, persistent shock, prolonged invasive mechanical ventilation, active malignancy with an estimated life expectancy < 1 year, end-stage renal disease (ESRD) and/or chronic dialysis, refractory heart failure, uncontrolled infection (such as perivalvular abscess or persistent bacteremia), or device-related IE that required urgent extraction. All other patients were classified as RW patients.

### 2.6. QIs and Guideline Adherence

QIs were based on international guideline recommendations and included four indicators: (1) adequate blood cultures (≥2 sets obtained before antibiotics), (2) TEE performed when indicated according to guideline-based diagnostic criteria, (3) appropriate antimicrobial therapy (guideline-concordant initial antibiotics with adequate duration based on microbiological data when available), and (4) multidisciplinary evaluation (heart team), followed by cardiac surgery when indicated (Class I/IIa) or documentation of contraindication [1,3,4,23]. For each patient, we computed a guideline adherence score (0–4) as the sum of fulfilled indicators. We then defined overall guideline adherence as achieving a pre-specified threshold of ≥3 out of 4 indicators.

## 3. Statistical Analysis

Continuous variables were described as mean ± standard deviation (SD) or median (interquartile range), depending on their distribution. Group comparisons used Student’s *t*-test or the Mann–Whitney U test for continuous data and the χ^2^ test or Fisher’s exact test for categorical data. Univariable analyses identified factors associated with the composite outcome and in-hospital mortality. Variables with *p* < 0.10 in univariable analysis, along with clinically relevant covariates, were entered into the multivariable model. Variables closely related to outcome components or those that showed collinearity with dominant severity markers were excluded during model optimization to preserve stability and avoid overfitting. Analyses were performed using available-case data, and only variables with sufficient completeness (>90% availability) and clinical reliability were included in multivariable models. Variables with higher missingness were excluded to preserve model stability. Results are presented as odds ratios with 95% confidence intervals. Model performance was assessed with the Hosmer–Lemeshow test and receiver operating characteristic (ROC) curves, and we examined collinearity through variance inflation factors. Comparisons between TE and RW patients, as well as between the two-time intervals (P1: 2011–2016 vs. P2: 2017–2025), included baseline characteristics, QIs, and outcomes. For the composite endpoint and mortality, adjusted analyses used logistic regression models that included age, sex, comorbidity burden, pathogen, and prosthetic or device-related status. All tests were two-sided, and *p* < 0.05 was considered statistically significant. All statistical analyses used R version 4.4.0 (2024-04-24 ucrt), “Puppy Cup” [24].

## 4. Results

### 4.1. Overall Cohort Characteristics

**Figure 1:**
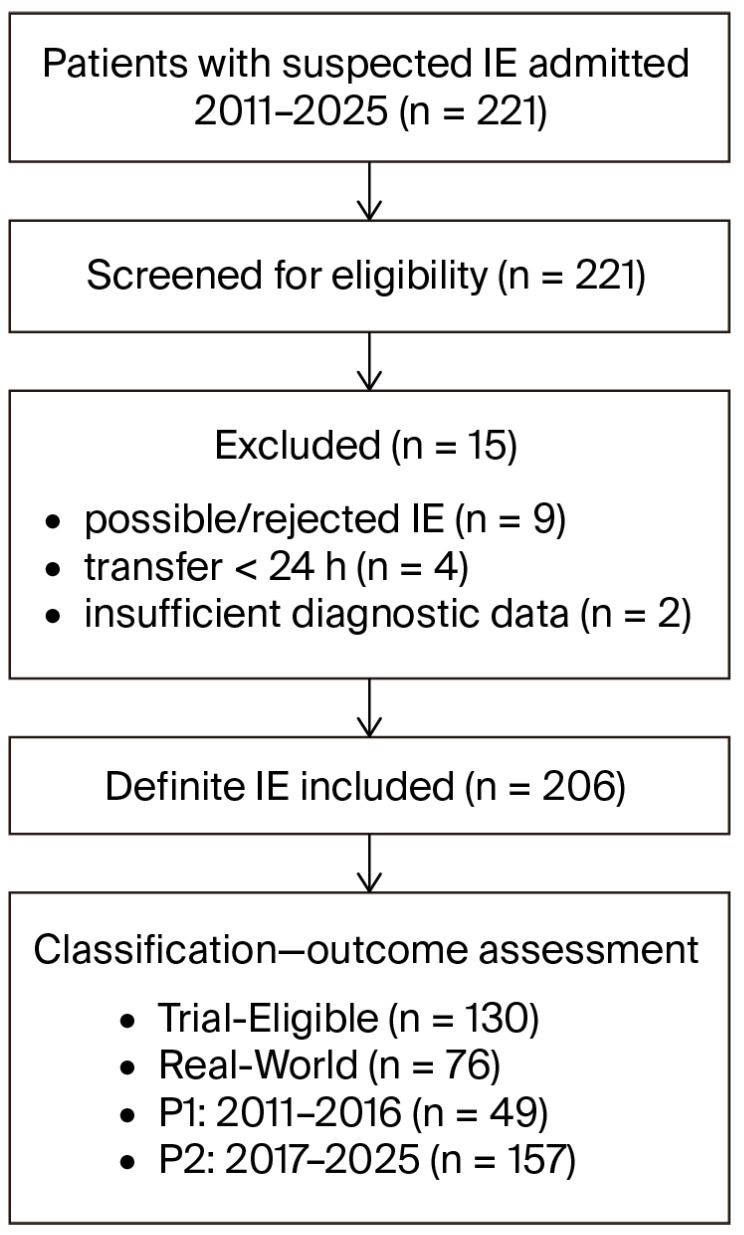
Study selection process. Legend: IE—infective endocarditis, n—number, h—hours, P1—period 1, P2—period 2.

Between 2011 and 2025, 221 patients were admitted with suspected IE. After diagnostic verification and exclusion of non-definite cases, early transfers, and incomplete records, 206 adults with definite IE were included. Of these, 130 (63.1%) met predefined trial eligibility criteria and were classified as TE, whereas 76 (36.9%) represented RW patients. Temporal stratification identified 49 patients in Period 1 (2011–2016) and 157 in P2 (2017–2025). The study flow is illustrated in Figure 1.

The overall cohort of 206 patients had a mean age of 63.0 ± 14.8 years, and 70.4% were male. Blood cultures were positive in 64.1%, and *Staphylococcus aureus* was identified in 14.1%. Vegetations were documented in 72.8%, and cardiac surgery was performed in 26.2%. Clinical severity was substantial. Sepsis at admission occurred in 25.2%, septic shock in 8.3%, and CS in 15.5%. The composite endpoint occurred in 61.6%, and in-hospital mortality was 32.5%. Missing data were limited for core clinical and outcome variables (<5%) but were higher for specific domains. These included complete SOFA components (~15%) and selected echocardiographic parameters, mainly TEE-related measures, which were missing in up to 20% of cases in P1, with substantial improvement in P2. The culture-negative rate (35.9%) was largely explained by prior antibiotic exposure before blood culture collection and less systematic microbiological sampling in P1. Baseline characteristics are summarized in Table 1.

### 4.2. Comparison Between TE and RW Patients

RW patients were older than TE patients (68.3 ± 12.1 vs. 59.9 ± 15.4 years, *p* < 0.001), which represents an absolute difference of approximately eight years. Male sex predominated overall but was more frequent in the TE cohort (78.5% vs. 56.6%, *p* = 0.002). The most common cardiovascular comorbidities were similarly distributed. HTN, DM, chronic HF, chronic obstructive pulmonary disease (COPD), and liver cirrhosis did not differ significantly between groups. CKD was more frequent in RW patients (34.2% vs. 18.5%, *p* = 0.01), while malignancy occurred exclusively in RW patients, which reflects eligibility criteria. Infection location was predominantly left-sided in both groups (>95%). Device-related endocarditis showed a clear imbalance. Cardiac implantable electronic device (CIED)-related IE was more common in RW patients (26.3% vs. 9.2%, *p* = 0.001), and transcatheter aortic valve implantation (TAVI)-associated IE occurred only in this cohort. Microbiological patterns were broadly comparable, although *Enterococcus* spp. were more frequent in RW patients (21.1% vs. 7.7%, *p* = 0.01). Culture-negative cases were similarly distributed. Echocardiographic findings were largely similar, except for a higher rate of perivalvular abscess in TE patients (27.7% vs. 13.2%, *p* = 0.02). Vegetation size and ventricular systolic function did not differ. Markers of acute severity were more prominent in RW patients. Sepsis at admission occurred twice as often (38.2% vs. 17.7%, *p* = 0.002), and septic shock was markedly more frequent (19.7% vs. 1.5%, *p* < 0.001). CS and acute heart failure showed no significant differences. Clinical outcomes were similar between groups for the composite endpoint and in-hospital mortality. However, major embolic events were more frequent in RW patients (35.5% vs. 20.8%, *p* = 0.02). Length of stay did not differ significantly (12.6 ± 11.1 vs. 13.5 ± 10.2 days, *p* = 0.331). RW and TE patient characteristics are summarized in Table 1.

### 4.3. Temporal Comparison Between Study Periods (P1 vs. P2)

Comparisons between study periods are presented in Table 2. The later cohort included a higher proportion of RW patients (*p* = 0.002), which indicates a shift toward more complex clinical profiles over time. Age and sex distribution remained stable. The high proportion of culture-negative IE in Period 1 (65.3% vs. 26.7% in P2) was primarily related to prior antibiotic exposure before blood culture collection and less systematic microbiological sampling. Testing for fastidious or fungal pathogens was not performed consistently in the earlier period. Device-related infections increased in P2, particularly CIED-associated cases. Diagnostic practices also evolved. TEE was used more frequently in the later period (*p* = 0.01). Larger vegetations were more often documented in P1, whereas embolic events were significantly more common in P2. This pattern suggests that embolic risk in the overall cohort was influenced by factors beyond vegetation size, including greater clinical severity and more systematic diagnostic assessment. Markers of clinical severity were more prevalent in P2, with higher rates of septic shock and CS. The composite endpoint increased (66.8% vs. 42.9%, *p* = 0.002), mainly due to septic complications and CS or acute HF. In-hospital mortality remained unchanged. Length of stay increased significantly in P2 (14.4 ± 11.5 vs. 8.2 ± 6.0 days, *p* = 0.002).

### 4.4. Predictors of Outcomes (Univariable and Multivariable Analysis)

In univariable analysis, markers of acute clinical severity, including sepsis at admission, shock states, renal replacement therapy, and impaired LVEF < 50%, showed the strongest association with the composite endpoint. *Staphylococcus aureus* infection showed a higher event rate, but it did not retain independent significance after multivariable adjustment. Perivalvular abscess showed a borderline association with the composite endpoint in univariable analysis (OR 2.2, 95% CI 1.01–4.9, *p* = 0.048) and was therefore interpreted as a trend rather than a robust independent predictor. CS and acute heart failure were associated with the composite endpoint in univariable analysis, but they were not retained in the final multivariable model because they did not provide independent predictive value after adjustment for sepsis at admission and LVEF, and they overlapped with components of the composite endpoint (Table 3). Variables that met statistical or clinical relevance criteria were entered into a multivariable logistic regression model. The final model confirmed sepsis at admission as the strongest independent predictor, followed by reduced LVEF < 50% and *streptococcal* infection, while prosthetic or device-related IE showed a protective trend (Table 4).

**Figure 2:**
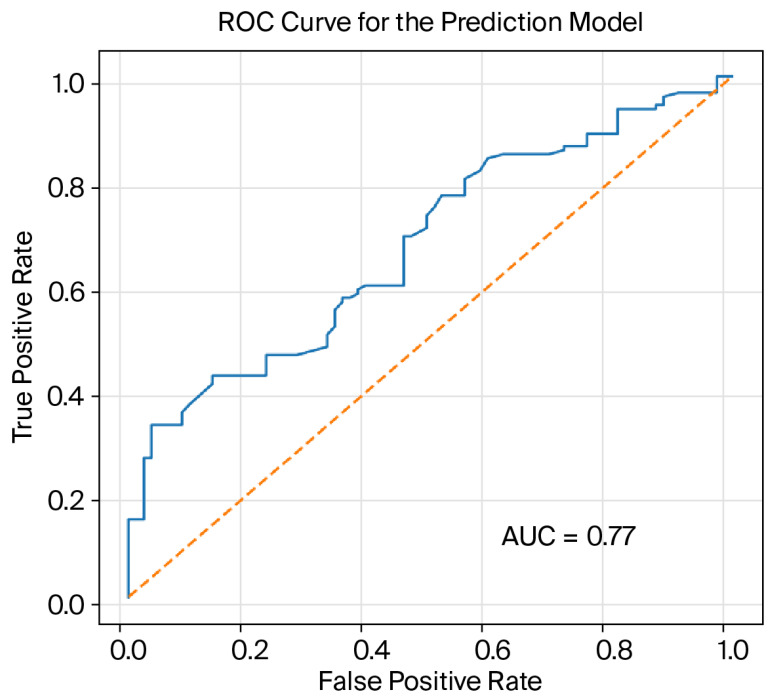
Receiver operating characteristic curve for the prediction model. Legend: AUC = area under the curve, ROC—receiver operating characteristic curve.

**Figure 3:**
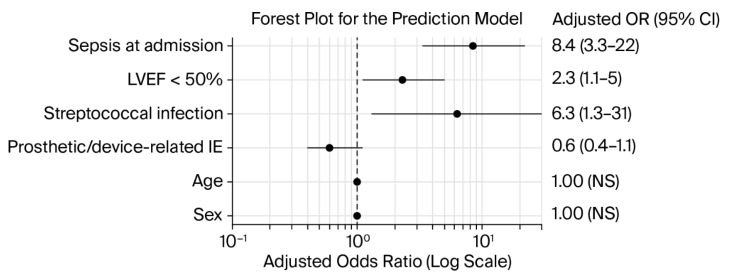
Adjusted predictors of embolic events derived from multivariable logistic regression models. Legend: LVEF = left ventricular ejection fraction, IE = infective endocarditis, OR = odds ratio, CI = confidence interval. Points indicate adjusted OR, horizontal lines show 95%, dashed vertical line denotes OR = 1, NS—non-significant.

The final prediction model, based on routinely available admission variables, showed acceptable discrimination (AUC 0.77) and good calibration. Figure 2 and Figure 3 illustrate the performance and structure of the prediction model. Figure 2 shows the ROC curve and demonstrates acceptable discrimination (AUC 0.77). Figure 3 presents the multivariable model as a forest plot and highlights the independent contribution of sepsis at admission, reduced LVEF, and microbiological factors.

### 4.5. QIs and Guideline Adherence

Quality of care improved over time. The proportion of patients who achieved a QI score ≥ 3 increased from 18.3% in P1 to 25.4% in P2 (OR 1.63, *p* = 0.32). A similar trend was observed for higher attainment in TE vs. RW patients (26.9% vs. 18.4%, OR 2.23, *p* = 0.05). Marked improvements were observed in key diagnostic and therapeutic processes: adequate blood cultures increased from 34.7% to 73.2% (OR 5.14, *p* < 0.001); TEE when indicated increased from 18.3% to 51.5% (OR 4.72, *p* = 0.001); appropriate antimicrobial therapy increased from 32.7% to 73.9% (OR 5.86, *p* < 0.001); and surgery when indicated increased from 12.2% to 30.5% (OR 3.18, *p* = 0.01). Differences between TE and RW patients were modest for individual indicators and were not statistically significant (Table 5).

### 4.6. Special Subgroup of Patients

#### 4.6.1. Subgroup Prosthetic/Device-Related IE:

Prosthetic valve IE was present in 81 of 206 patients (39.3%), including eight TAVI-associated cases (3.9%). CIED-related IE was identified in 32 patients (15.5%) (Table 1). There was overlap between categories: 17 patients had both prosthetic valve and CIED-related IE, and seven TAVI cases also had CIED-related IE. Among patients with prosthetic valve IE, in-hospital mortality was 18.5% (15/81), the composite endpoint occurred in 51.9% (42/81), and surgery was performed in 32.1% (26/81). The dominant microbiological categories were culture-negative IE or unknown pathogen at 37.0% (30/81), *Enterococcus faecalis* at 18.5% (15/81), *Staphylococcus aureus* at 11.1% (9/81), and other pathogens at 14.8% (12/81). In CIED-related IE, mortality was 28.1% (9/32), the composite endpoint occurred in 53.1% (17/32), and surgery was performed in 28.1% (9/32). The most frequent pathogens were *Staphylococcus aureus* at 28.1% (9/32), *Enterococcus faecalis* at 15.6% (5/32), other pathogens at 15.6% (5/32), and culture-negative IE or unknown pathogen at 15.6% (5/32). Among TAVI-associated IE cases, mortality was 37.5% (3/8), the composite endpoint occurred in 50.0% (4/8), and surgery was performed in 50.0% (4/8). Pathogens were culture-negative or unknown in 62.5% (5/8), *Staphylococcus aureus* in 25.0% (2/8), and *Enterococcus faecalis* in 12.5% (1/8). Due to overlap between categories and the dataset structure, fully stratified outcome rates could not be calculated consistently for all subgroups. 

#### 4.6.2. Subgroup On-Site Cardiac Surgery Program

Cardiac surgery was performed in 30 patients before 2023 and in 24 patients after implementation of the local surgical program (total 26.2%), which corresponded to an increase in the surgical rate from 18% to 57% in the post-implementation subgroup. The relatively low rate of cardiac surgery reflects limited local availability in the earlier period; all patients were referred to tertiary centers or were deemed inoperable due to advanced clinical severity and comorbidities. Use of TEE also increased (71% vs. 41%), and post-implementation cases showed a higher proportion of severe presentations, including sepsis at admission (45% vs. 20%). Despite this more complex case mix, in-hospital mortality was numerically lower after 2023 (21% vs. 35%), whereas the composite endpoint remained frequent (67% vs. 58%). Given the limited number of cases treated after program initiation and the initially low surgical volume, these findings should be interpreted as exploratory.

## 5. Discussion

To our knowledge, this is the first long-term study from Eastern Europe to jointly analyze RW IE characteristics, RCT eligibility, guideline adherence, and temporal outcome trends in a county hospital setting. Several key findings underscore the utility and practical significance of our research. First, the cohort illustrates increasing clinical complexity in IE, with a substantial proportion of patients who present characteristics not typically represented in randomized trials. Second, adherence to guideline-based QIs improved significantly over time. Third, despite these improvements in process-of-care measures, the incidence of major in-hospital complications increased, reflecting a shift toward a more severe case mix rather than a decline in the quality of care. The demographic and clinical differences between TE and RW patients resemble patterns described in large observational registries, where aging populations, device-related infections, and comorbidity burden have reshaped IE epidemiology [6,7,25,26,27]. Only a subset of patients (63.1%) met predefined criteria that approximated enrollment in contemporary randomized IE trials. This observation parallels analyses of trial cohorts such as the Partial Oral Treatment of Endocarditis (POET), where strict inclusion criteria systematically exclude older, unstable, or highly comorbid patients [9,10]. Similar discrepancies have been reported in registry comparisons, where RW populations demonstrate higher complication rates than trial cohorts [25,26,27,28,29].

The ESC-EURObservational Research Programme (EORP) European Endocarditis (EURO-ENDO) registry (ESC-EORP EURO-ENDO), a large prospective cohort that includes over 3000 patients with IE from 156 centers across 40 countries, provides the most comprehensive contemporary data on epidemiology, management, and outcomes. In-hospital mortality was reported at approximately 17%, with notable representation of staphylococcal, enterococcal, prosthetic valve, and device-related infections [30,31,32]. In contrast, in-hospital mortality in our cohort (32.5%) appears higher than that reported in the EURO-ENDO registry but remains within the range observed in contemporary high-severity RW series (approximately 25–30%) and is comparable to prior Romanian hospital cohorts that report mortality approaching one-third of cases [16,17,18,26,30,31,32,33]. This difference is largely explained by case mix and clinical profile at presentation. A substantial proportion of our RW patients presented with sepsis or shock, which are conditions consistently identified as dominant short-term mortality drivers in IE [22,26,34,35]. In addition, our cohort reflects the ongoing epidemiologic transition toward older and more comorbid populations with a higher burden of prosthetic or device-related infections. These patterns are associated with increased early clinical risk in observational studies [30,33]. Finally, regional system factors, particularly the timing and feasibility of surgery or device extraction, may influence early outcomes. A cardiovascular surgery program at our hospital began in 2023. Observational data consistently demonstrate improved survival in high-risk subgroups when timely surgical management is achieved [36,37]. Our findings underscore the importance of interpreting outcome metrics within RW epidemiologic and organizational frameworks.

The temporal comparison between the P1 and P2 periods in our cohort reveals a pattern that is increasingly recognized in contemporary IE research: advances in structured care occur alongside a rising burden of clinical complexity. Patients in the later period (P2) showed significantly higher adherence to QIs outlined in ESC guidelines, including broader use of TEE, routine microbiological sampling, and multidisciplinary evaluation. These improvements likely enhanced diagnostic precision and improved consistency in clinical pathways [23,30,33]. The reduction in culture-negative IE over time likely reflects better adherence to guideline-recommended microbiological protocols, including earlier blood culture acquisition and more structured diagnostic workflows. Residual culture-negative cases may still relate to prior antibiotic use and limited access to advanced microbiological testing in routine practice. At the same time, the P2 group included a greater proportion of device-associated infections and septic presentations, both of which are independently linked to more severe clinical courses and higher complication rates in registry analyses [30,33]. Prosthetic and device-related IE accounted for a large proportion of cases (96/206) and was associated with higher clinical complexity and a microbiological shift toward enterococci and *Staphylococcus aureus*. Although these subgroups showed substantial rates of complications and mortality, their independent prognostic impact was attenuated after adjustment, which indicates that early outcomes are primarily driven by acute clinical severity rather than infection substrate alone. The observation that in-hospital mortality remained relatively stable despite an increase in serious complications suggests that improvements in care processes may have partially mitigated the impact of rising biological severity. This contrast highlights that adherence metrics primarily reflect quality of care, whereas short-term outcomes remain strongly influenced by baseline disease burden. Importantly, this temporal pattern mirrors differences observed between TE and RW populations: process adherence is necessary but insufficient to overcome the prognostic weight of acute severity markers. These longitudinal findings align with European registry data, which show that coordinated diagnostic and management pathways are associated with improved process measures and more consistent early care [32,33].

Multivariable analysis identified sepsis at presentation, LVEF < 50%, and streptococcal infection as independent predictors of the composite in-hospital endpoint. This pattern aligns with established prognostic frameworks in IE, where the acute systemic inflammatory response and cardiac functional reserve act as stronger determinants of early outcomes than baseline demographic characteristics. The persistence of microbiological category as an independent factor suggests that pathogen-specific virulence and tissue invasion significantly influence complication risk. In contrast, age and sex did not retain independent predictive value after adjustment, which indicates that these variables primarily serve as background vulnerability markers rather than direct drivers of short-term events. Similarly, the apparent attenuation of risk associated with prosthetic or device-related infection and certain microbiological subtypes, such as staphylococcal infection in our study, should not be interpreted as biological protection. Instead, once dominant severity markers such as sepsis and ventricular dysfunction are accounted for, contextual characteristics lose independent statistical significance. This finding reflects model adjustment within a heterogeneous clinical population rather than a reduction in clinical risk. Variables marked with dashes in Table 3, including CS, acute heart failure, and perivalvular abscess, were examined but not retained in the final model because they did not add independent predictive information after adjustment for overlapping severity indicators. Their exclusion represents statistical optimization and reflects statistical collinearity and overlap with outcome components rather than clinical relevance, as these features remain integral components of the composite endpoint and overall disease severity. Other clinically relevant variables were evaluated but not included in the final multivariable model because they did not provide independent predictive value after adjustment for dominant severity markers or were not consistently available in the retrospective dataset, such as referral delay and oral health status. Inclusion of highly correlated variables may reduce model stability and lead to overfitting, particularly in single-center cohorts with a limited number of events. We therefore prioritized variables that were systematically recorded and clinically interpretable at admission, which allowed construction of a robust model suitable for RW application. Importantly, the multivariable findings align with descriptive cohort patterns. The same markers that differentiated TE from RW patients, particularly sepsis and impaired ventricular function, were those most strongly associated with adverse outcomes. From a practical perspective, the identified predictors are readily available at admission and therefore useful for early risk stratification. Recognition of sepsis and ventricular dysfunction should prompt intensified monitoring, rapid antimicrobial optimization, and early surgical assessment when appropriate. The model demonstrated acceptable discrimination (AUC 0.77), comparable to performance reported in observational IE prognostic studies, which supports its applicability to RW clinical decision-making rather than highly selected trial populations [38,39,40,41].

Our study demonstrates a substantial temporal improvement in key QIs of IE management, particularly in microbiological diagnosis, systematic use of TEE, and implementation of guideline-directed antimicrobial therapy. These changes likely reflect increased adherence to ESC recommendations and improved local diagnostic infrastructure, including broader access to TEE after 2016. Importantly, differences between TE and RW patients were limited, which indicates that improvements in care delivery extended beyond selected lower-risk populations. The marked increase in blood culture adequacy and TEE utilization is clinically relevant, as both are strongly linked to diagnostic accuracy, embolic risk stratification, and appropriate surgical referral. Similarly, the higher proportion of patients who received appropriate antimicrobial therapy and surgery when indicated suggests better integration of cardiology, infectious diseases, and cardiac surgery teams. Taken together, these findings support a progressive alignment of RW practice with guideline-recommended care pathways, while they highlight a persistent gap in achieving high composite QI scores in all patients.

Although the local cardiac surgery program became operational only in December 2023 and the number of IE procedures remained limited at first, the proportion of operated patients increased substantially (30 before 2023 vs. 24 after 2023), which suggests improved access to timely surgical management. The availability of on-site cardiac surgery was associated with a higher rate of surgical treatment and more frequent use of advanced imaging, which suggests improved implementation of guideline-based management. Patients treated after 2023 presented with more severe clinical profiles, which is consistent with a shift toward managing more complex cases locally. The observed numerical reduction in mortality, despite greater disease severity, supports a potential benefit of improved access to surgery and multidisciplinary decision-making. However, because relatively few IE cases were operated on during the early phase of the program, this comparison should be considered hypothesis-generating. Larger post-implementation cohorts will be needed to confirm whether local surgical availability translates into sustained improvement in outcomes.

Overall, this county-level cohort reflects the epidemiologic transition of IE described in contemporary registries, which is characterized by older patients with more comorbidities and an increasing prevalence of prosthetic and CIED-related infections. While the microbiological patterns align with international data, the greater clinical severity observed in a regional hospital setting highlights the heterogeneity of RW IE beyond tertiary referral centers. These findings emphasize that regional cohorts provide essential complementary insights into disease burden, care delivery, and complication patterns. Therefore, it is crucial to interpret outcomes within this broader clinical and organizational context when applying registry and trial data to everyday clinical practice.

## 6. Study Strengths and Limitations

This study has several strengths. Consecutive patient inclusion based on standardized diagnostic criteria reduced selection bias and ensured internal consistency. The integration of clinical, microbiological, imaging, and process-of-care variables allowed a comprehensive evaluation of IE, while the combined assessment of temporal trends, trial eligibility, guideline adherence, and outcomes provided a multidimensional perspective. Independent case adjudication further supported data reliability. Limitations should also be considered. The retrospective single-center design exposes the analysis to potential information bias, residual confounding, and limited external generalizability. Temporal differences may partly reflect changes in referral patterns and case mix. The prediction model was internally derived and was not externally validated; therefore, results should be interpreted as exploratory associations rather than causal effects. Some clinically relevant variables were excluded from multivariable analysis due to collinearity or instability, and certain endpoints relied on routine clinical documentation, which may have introduced classification variability. The prediction model therefore represents a pragmatic risk stratification approach based on variables available at admission, and it highlights the potential incremental value of future datasets that incorporate referral timing, frailty, or additional biological markers.

## 7. Future Directions

Future research should focus on multicenter validation of process-based QIs and prospective assessment of risk stratification in routine practice. Stronger multidisciplinary care pathways and interoperable digital registries that integrate imaging, microbiological, and outcome data may improve clinical decision-making and benchmarking across centers. Particular attention should be given to underrepresented populations in clinical trials, especially older patients and those with device-related IE, to better align management strategies with RW complexity.

## 8. Conclusions

RW IE is characterized by a high complication burden and a persistent trial-eligibility gap. Although adherence to guideline-based care improved over time, increasing clinical severity likely reduced potential gains in outcomes and highlights the need for pragmatic, multidisciplinary management strategies adapted to RW patient complexity.

Key Messages

- RW IE patients differ substantially from RCT populations.
- Guideline adherence improved over time.
- The complication burden increased due to greater severity.
- Mortality remained stable despite higher clinical complexity.
- Early severity markers predict in-hospital outcomes.
- Structured care pathways support consistent management.
- ESC QI adherence is achievable in regional hospitals.
- Regional registries complement tertiary sources of evidence.

## Figures and Tables

**Table 1:** Baseline characteristics, management, and in-hospital outcomes of patients with definite IE, stratified by trial eligibility.

Variable	Overall (n = 206)	Trial-Eligible (n = 130)	Real World (n = 76)	*p*-Value (95% CI)
Demographics				
Age, years (mean ± standard deviation)	63.0 ± 14.8	59.9 ± 15.4	68.3 ± 12.1	<0.001 (−12.2 to −4.6)
Male sex, n (%)	145 (70.4%)	102 (78.5%)	43 (56.6%)	0.002. (3.5 to 39.3)
Comorbidities				
Hypertension, n (%)	125 (60.7%)	79 (60.8%)	46 (60.5%)	1.000
Diabetes mellitus, n (%)	37 (18.0%)	24 (18.5%)	13 (17.1%)	0.955
CKD (≥stage 3), n (%)	47 (22.8%)	21 (18.5%)	26 (34.2%)	0.01 (4.9 to 28.6)
COPD, n (%)	17 (8.3%)	8 (6.2%)	9 (11.8%)	0.242
IVDU, n (%)	2 (1.0%)	0 (0.0%)	2 (2.6%)	0.135
Chronic HF, n (%)	139 (67.5%)	86 (66.2%)	53 (69.7%)	0.707
Liver cirrhosis, n (%)	5 (2.42%)	2 (2.6%)	3 (2.3%)	1.000
Malignancy, n (%)	33 (16.0%)	0 (0.0%)	33 (43.4%)	0.0001 (30 to 54.6)
Infection location				
Left-sided IE, n (%)	197 (95.6%)	125 (96.2%)	72 (94.7%)	0.728
Right-sided IE, n (%)	9 (4.4%)	5 (3.8%)	4 (5.3%)	0.718
Prosthetic IE, n (%)	81 (%)	54 (41.5%)	27 (35.5%)	0.394
CIED-related IE, n (%)	32 (15.5%)	12 (9.2%)	20 (26.3%)	0.001 (2.3 to 31.8)
TAVI-associated IE	8 (3.8%)	0 (0.0%)	8 (10.5%)	<0.001 (2.6 to 19.4)
Microbiology				
*S. aureus*, n (%)	29 (14.1%)	22 (16.9%)	7 (9.2%)	0.184
*Enterococcus* spp., n (%)	26 (12.6%)	10 (7.7%)	16 (21.1%)	0.010 (0.2 to 27.3)
Viridans streptococci, n (%)	20 (9.7%)	18 (13.8%)	2 (2.6%)	0.009 (−20.1 to 0.2)
Culture-negative IE, n (%)	74 (35.9%)	45 (34.6%)	29 (38.1%)	0.480
Echocardiography				
Vegetation present, n (%)	150 (72.8%)	93 (71.5%)	57 (75.0%)	0.590
Vegetation >10 mm, n (%)	95 (46.1%)	60 (46.2%)	35 (46.1%)	0.989
Perivalvular abscess, n (%)	46 (22.3%)	36 (27.7%)	10 (13.2%)	0.02 (1.8 to 28.6)
Severe valvular regurgitation, n (%)	79 (38.3)	53 (40.8%)	26 (34.2%)	0.350
LVEF < 50%, n (%)	93 (45.1%)	61 (46.9%)	32 (42.1%)	0.599
Clinical presentation/severity				
Acute HF on admission, n (%)	25 (12.1%)	12 (9.2%)	13 (17.1%)	0.147
Sepsis at admission, n (%)	52 (25.2%)	23 (17.7%)	29 (38.2%)	0.002 (−37.3 to −2.9)
Septic shock, n (%)	17 (8.3%)	2 (1.5%)	15 (19.7%)	0.0001 (−29.6% to −6.9%)
Cardiogenic shock, n (%)	32 (15.5%)	15 (11.5%)	17 (22.4%)	0.061 (3.7 to 25.8)
Outcomes				
Composite endpoint, n (%) *	126 (61.6%)	75 (57.6%)	51 (67.1%)	0.218
In-hospital mortality, n (%)	67 (32.5%)	37 (28.5%)	30 (39.5%)	0.141
Renal replacement therapy (acute)	8 (3.8%)	6 (4.6%)	2 (2.6%)	0.713
Any embolic event, n (%) **	54 (26.2%)	27 (20.8%)	27 (35.5%)	0.02 (−2.8 to 32.1)
Length of stay, days (mean ± standard deviation)	13.05 ± 10.6	12.6 ± 11.1	13.5 ± 10.2	0.331

Legend: CKD = chronic kidney disease was defined as estimated glomerular filtration rate < 60 mL/min/1.73 m^2^ (stage ≥ 3) or need for chronic dialysis, Chronic HF = existing heart failure documented before the index hospitalization, COPD = chronic obstructive pulmonary disease, IE = infective endocarditis, IVDU = intravenous drug use, CIED = cardiac implantable electronic device, TAVI = transcatheter aortic valve implantation, LVEF = left ventricular ejection fraction, Acute HF on admission = acute decompensated heart failure present at hospital admission, 95% CI = 95% confidence intervals, *p*-value < 0.05 was considered statistically significant, * composite in-hospital endpoint = occurrence of at least one of the following events: acute heart failure requiring intravenous therapy or ventilatory support, shock (cardiogenic and/or septic), embolic events symptomatic or confirmed by imaging or surgery, severe infectious complications (such as abscess, fistula, or metastatic infection), renal replacement therapy, and all-cause in-hospital mortality. ** cerebral asymptomatic imagistic detected or symptomatic, splenic, renal, hepatic, mesenteric, or limb arteries.

**Table 2:** Comparison between study periods (P1: 2011–2016 vs. P2: 2017–2025), including trial-eligibility distribution, quality indicators, and outcomes.

Section/Variable	P1 (2011–2016)	P2 (2017–2025)	*p*-Value 95% CI
Cohort composition	49	157	
Trial-eligible (TE)	40 (81.6%)	90 (57.3%)	0.002 (−40.5 to −3.8)
Real-world (RW)	9 (18.4%)	67 (42.7%)	0.002 (3.8 to 40.5)
Demographics			
Age, years (mean ± standard deviation)	62.0 ± 13.3	63.4 ± 15.3	0.563
Male sex, n (%)	39 (79.6%)	106 (67.5%)	0.151
Comorbidities			
Hypertension	34 (69.4%)	91 (58.0%)	0.153
Diabetes mellitus	7 (14.3%)	30 (19.1%)	0.443
Coronary artery disease	11 (22.4%)	43 (27.4%)	0.492
Chronic HF, n (%)	43 (87.8%)	96 (61.1%)	<0.001 (−38.5 to −14.7)
Atrial fibrillation	14 (28.6%)	59 (37.6%)	0.250
CKD (≥stage 3), n (%)	8 (16.3%)	39 (24.8%)	0.01 (12.6 to 28.9)
Liver cirrhosis, n (%)	1 (2.1%)	4 (2.5%)	0.983
Malignancy, n (%)	4 (8.2%)	29 (18.5%)	0.086 (−6.0 to 22.0)
IVDU	0 (0.0%)	2 (1.2)	0.275
Infection location			
Prosthetic valve IE, n (%)	21 (42.8%)	60 (38.2)	0.384
CIED-related IE, n (%)	1 (2.0%)	31 (19.7%)	0.003 (3.6 to 26.3)
TAVI-associated IE, n (%)	0 (0.0%)	8 (5.1%)	0.202
Microbiology			
*S. aureus*, n (%)	6 (12.2%)	23 (14.6%)	0.262
*Enterococcus* spp., n (%)	4 (8.1%)	22 (14.01%)	0.275
Viridans streptococci, n (%)	2 (4.08)	18 (11.4%)	0.126
Culture-negative IE, n (%)	32 (65.3%)	42 (26.7%)	0.001 (−43.1 to −12.4)
Echocardiography			
TEE performed, n (%)	9 (18.3%)	81 (51.5%)	0.01 (−1.1 to 38.9)
Vegetation >10 mm, n (%)	42 (85.7%)	53 (33.8%)	<0.001 (−66.1 to −31.9)
Perivalvular abscess, n (%)	4/49 (8.2%)	42 (26.8%)	0.006 (8.3 to 28.9)
Severe regurgitation, n (%)	25 (51.0%)	54 (34.4%)	0.05 (−32.5 to −0.8)
LVEF < 50%	30 (61.2%)	63 (40.1%)	0.010 (−40.8 to 0.7)
Clinical presentation/severity			
Acute HF on admission, n (%)	7 (14.2%)	18 (11.4%)	0.317.
Sepsis at admission, n (%)	9 (18.3%)	43 (27.3%)	0.07 (15.8 to 37.7)
Septic shock, n (%)	2 (4.08%)	15 (9.5%)	0.02 (3.0 to 14.2)
Cardiogenic shock, n (%)	3 (6.1%)	29 (18.4%)	0.01 (2.8 to 28.7)
Outcomes			
Composite endpoint, n (%) *	21 (42.9%)	105 (66.8%)	0.002 (3.1 to 44.3)
In-hospital mortality, n (%)	17 (34.7%)	50 (31.8%)	0.844 (−18.0 to 12.3)
Renal replacement therapy (acute), n (%)	4 (8.2%)	4 (2.5%)	0.09 (−18.2 to 3.1)
Any embolic event, n (%) **	4 (8.2%)	50 (31.8%)	<0.001 (5.9 to 36.3)
Length of stay, days (mean ± standard deviation)	8.2 ± 6.0	14.4 ± 11.5	0.002 (3.6 to 8.6)

Legend: CKD = chronic kidney disease was defined as estimated glomerular filtration rate < 60 mL/min/1.73 m^2^ (stage ≥ 3) or need for chronic dialysis, Chronic HF = existing heart failure documented before the index hospitalization, IE = infective endocarditis, IVDU = intravenous drug use, CIED = cardiac implantable electronic device, TAVI = transcatheter aortic valve implantation, TEE = transesophageal echocardiography, LVEF = left ventricular ejection fraction, Acute HF on admission = acute decompensated heart failure present at hospital admission, 95% CI = 95% confidence intervals. *p*-value < 0.05 was considered statistically significant. * composite in-hospital endpoint = occurrence of at least one of the following events: acute heart failure requiring intravenous therapy or ventilatory support, shock (cardiogenic and/or septic), embolic events symptomatic or confirmed by imaging or surgery, severe infectious complications (such as abscess, fistula, or metastatic infection), renal replacement therapy, and all-cause in-hospital mortality. ** cerebral asymptomatic imagistic detected or symptomatic, splenic, renal, hepatic, mesenteric, or limb arteries.

**Table 3:** Univariable logistic regression for the composite in-hospital endpoint.

Variable	Univariable OR	95% CI	*p*-Value
Sepsis at admission	9.1	3.8–21.5	<0.001
Streptococcal infection	5.8	1.4–24.0	0.014
Cardiogenic shock	3.9	1.8–8.4	<0.001
Acute heart failure	2.7	1.4–5.0	0.002
LVEF < 50%	2.6	1.3–5.2	0.006
Perivalvular abscess	2.2	1.0–4.9	0.048
*Staphylococcus aureus* infection	1.8	0.9–3.6	0.080
*Enterococcal* infection	1.4	0.7–2.9	0.320
Age (per 10 years)	1.02	0.99–1.04	0.160
Male sex	0.93	0.55–1.60	0.800
Prosthetic/device-related IE	0.70	0.40–1.20	0.190

Legend: OR = odds ratio; CI = confidence interval; IE = infective endocarditis; LVEF = left ventricular ejection fraction. Reference microbiological category: culture-negative IE. Values are ORs with 95% CIs obtained from univariable logistic regression assessing associations with the composite in-hospital endpoint (acute heart failure, cardiogenic shock, embolic events, renal replacement therapy, or in-hospital death). Variables with *p* < 0.10 and clinically relevant factors were considered for multivariable modeling.

**Table 4:** Multivariable logistic regression for the composite in-hospital endpoint.

Predictor	Adjusted OR (95% CI)	*p*-Value
Sepsis at admission	≈8.4 (3.3–21.8)	<0.001
LVEF < 50%	≈2.3 (1.1–5.0)	0.032
*Streptococcal* infection	≈6.3 (1.3–30.6)	0.024
Prosthetic/device-related IE	≈0.6 (0.4–1.1)	0.080
Age	≈1.0	NS
Sex	≈1.0	NS

Legend: OR = odds ratio; CI = confidence interval; IE = infective endocarditis; LVEF = left ventricular ejection fraction. Reference microbiological category: culture-negative IE.

**Table 5:** Extended quality-of-care indicators according to study period and trial eligibility.

Variable	P1 n (%)	P2 n (%)	OR (95% CI) P2 vs. P1	*p*	TE n (%)	RW n (%)	OR (95% CI) TE vs. RW	*p*
Overall patients (206), n	49	157	-	-	130	76	**-**	**-**
QI score ≥ 3	9 (18.3%)	40 (25.4%)	1.63 (0.62–4.27)	0.32	35 (26.9%)	14 (18.4%)	2.23 (0.96–5.19)	0.05
Adequate blood cultures, n (%) *	17 (34.7%)	115 (73.2%)	5.14 (2.47–10.7)	<0.001	81 (62.3%)	51 (67.1%)	0.80 (0.44–1.46)	0.588
TEE performed when indicated, n (%)	9 (18.3%)	81 (51.5%)	4.72 (2.10–10.6)	0.001	52 (40.0%)	38 (50.0%)	0.67 (0.37–1.21)	0.18
Appropriate antimicrobial therapy, n (%) **	16 (32.7%)	116 (73.9%)	5.86 (2.85–12.0)	<0.001	81 (62.3%)	51 (67.1%)	0.80 (0.44–1.46)	0.588
Surgery when indicated n (%)	6 (12.2%)	48 (30.5%)	3.18 (1.2–8.2)	0.01	35 (26.9%)	19 (25.0%)	1.10 (0.56–2.15)	0.890

Legend: QI = quality indicator; P1 interval (2011–2016), P2 interval (2017–2025), TE = trial-eligible; RW = real-world; TEE = transesophageal echocardiography; OR = odds ratio; CI = confidence interval, n = numbers. *p*-value < 0.05 was considered statistically significant, * ≥2 blood culture sets prior to antibiotics, ** guideline-concordant initial antibiotics combined with adequate duration antimicrobial therapy according to microbiological data when available.

## Data Availability

Data sharing is not applicable to this article as no datasets were generated or analyzed during the current outline of advances in our field of expertise.
